# Identifying deprived “slum” neighbourhoods in the Greater Accra Metropolitan Area of Ghana using census and remote sensing data

**DOI:** 10.1016/j.worlddev.2023.106253

**Published:** 2023-07

**Authors:** Robert MacTavish, Honor Bixby, Alicia Cavanaugh, Samuel Agyei-Mensah, Ayaga Bawah, George Owusu, Majid Ezzati, Raphael Arku, Brian Robinson, Alexandra M. Schmidt, Jill Baumgartner

**Affiliations:** a Department of Epidemiology, Biostatistics, and Occupational Health, McGill University, Montreal, Canada; b Institute for Health and Social Policy, McGill University, Montreal, Canada; c Institute of Public Health and Wellbeing, University of Essex, Colchester, England; d Department of Geography, McGill University, Montreal, Canada; e Department of Geography and Resource Development, University of Ghana, Accra, Ghana; f Institute of Statistical, Social and Economic Research, University of Ghana, Accra, Ghana; g Faculty of Medicine, School of Public Health, Imperial College, London, England; h Institute for Global Health, University of Massachusetts Amherst, Amherst, United States; i Department of Environmental Health Sciences, University of Massachusetts Amherst, Amherst, United States

**Keywords:** Informal settlements, Satellite imagery, Urban poverty

## Abstract

**Background:**

Identifying urban deprived areas, including slums, can facilitate more targeted planning and development policies in cities to reduce socio-economic and health inequities, but methods to identify them are often ad-hoc, resource intensive, and cannot keep pace with rapidly urbanizing communities.

**Objectives:**

We apply a spatial modelling approach to identify census enumeration areas (EAs) in the Greater Accra Metropolitan Area (GAMA) of Ghana with a high probability of being a deprived area using publicly available census and remote sensing data.

**Methods:**

We obtained United Nations (UN) supported field mapping data that identified deprived “slum” areas in Accra’s urban core, data on housing and population conditions from the most recent census, and remotely sensed data on environmental conditions in the GAMA. We first fitted a Bayesian logistic regression model on the data in Accra’s urban core (n=2,414 EAs) that estimated the relationship between housing, population, and environmental predictors and being a deprived area according to the UN’s deprived area assessment. Using these relationships, we predicted the probability of being a deprived area for each of the 4,615 urban EAs in GAMA.

**Results:**

899 (19%) of the 4,615 urban EAs in GAMA, with an estimated 745,714 residents (22% of its urban population), had a high predicted probability (≥80%) of being a deprived area. These deprived EAs were dispersed across GAMA and relatively heterogeneous in their housing and environmental conditions, but shared some common features including a higher population density, lower elevation and vegetation abundance, and less access to indoor piped water and sanitation.

**Conclusion:**

Our approach using ubiquitously available administrative and satellite data can be used to identify deprived neighbourhoods where interventions are warranted to improve living conditions, and track progress in achieving the Sustainable Development Goals aiming to reduce the population living in unsafe or vulnerable human settlements.

## 1 Introduction

Rapid urbanization has stressed urban infrastructure in low- and middle-income countries, forcing over one billion residents, including 238 million in sub-Saharan Africa, into deprived urban “slum” areas (UN-Habitat, 2016; UN-MDG, 2015). Such areas pose a grave challenge to urban sustainability and development due to spatially-determined health risks and vulnerabilities, including insecure tenure, unsafe living and environmental conditions, and crowding (Baker et al., 2016; Mberu et al., 2017; UN-Habitat, 2016). Compared with other city residents, people living in slums on average have worse health (Ezeh et al., 2017; Wanyama et al., 2019), poorer access to services and infrastructure, and larger barriers to economic opportunities (Bird et al., 2017; UN-Habitat, 2016).

The Sustainable Development Goal 11 (SDG-11) aims to “make cities and human settlements inclusive, safe, resilient and sustainable” by 2030, which includes slum upgrading (United Nations, 2020). Evaluating this objective requires that slums, informal settlements, and other areas of inadequate living conditions in cities be identified over time and across a range of settings using available data sources. Yet slums and other deprived urban areas are often not mapped accurately or routinely across global cities (Thomson et al., 2020). The lack of standardized methods to produce accurate, timely, and scalable maps of slums leave many of them hidden in the urban data systems used to inform policy and programming, including the monitoring of progress in meeting SDG-11 (Lilford et al., 2019).

Research during the past decade has produced a number of approaches to identify deprived ‘slum’ areas in cities (Thomson et al., 2020). Field-based approaches (i.e., community mapping) can produce accurate and context-specific maps, but are expensive and often limited in geographic scope (Ayson, 2018; Karanja, 2010; Livengood & Kunte, 2012; Muller & Mbanga, 2012). Other studies increase geographic coverage by using household-level survey or census data to identify households with features that match the United Nations Human Settlements Programme (UN-Habitat) slum criteria (i.e., absence of secure tenure, safe drinking water, adequate living space, hygienic sanitation facilities, or durable building materials) (Fink et al., 2014; Günther & Harttgen, 2012; Kyu et al., 2013; Patel et al., 2014). This approach identifies clusters of vulnerable households but can miss important neighbourhood factors (e.g., density, greenspace for play or relaxation, unsafe topography) (Engstrom et al., 2015). More recent studies try to better capture neighbourhood physical characteristics using visual or machine-learning classification of remote sensing data (Ibrahim et al., 2019; Kuffer et al., 2016; Kuffer et al., 2018; Verma et al., 2019; Williams et al., 2020), though these methods can miss important housing or social variables (e.g., tenure security, access to public services) (Owusu et al., 2021), and tend to overemphasize irregular settlement patterns which are often but not always a feature of slums (Ibrahim et al., 2019; Kuffer et al., 2018; Verma et al., 2019). What is needed are integrated and statistically robust approaches to mapping these areas that leverage available data and the combined strengths of current approaches to produce city-scale maps of deprived areas which are context specific and can be produced on a routine basis across multiple cities and countries (Thomson et al., 2020).

In this study, we developed and applied a Bayesian spatial modelling framework for identifying deprived “slum” areas (generally referred to as *deprived areas* hereafter) and their social and environmental features for the Greater Accra Metropolitan Area (GAMA). Our study makes both practical and methodological contributions to existing slum identification studies. We uniquely integrate publicly available census and remote sensing data to capture a range of social and physical features of homes and neighbourhoods, and extend the existing geographic coverage of deprived ‘slum’ area identification for Accra Metropolitan Area (AMA) to the rapidly growing suburban regions that surround it. Further, our novel use of a Bayesian spatial modeling approach naturally provides uncertainty estimates of the associations of housing and environmental metrics with deprived area classification, and can better handle the inclusion of latent spatial random effects to account for possible spatial structures that remain in the data after accounting for the available explanatory variables. Our study provides small enumeration area-level classification of deprived areas to capture and understand heterogeneity within neighbourhoods of low- and middle-income countries, and is scalable in nature to facilitate the use of our model in other geographical contexts. This study was conducted within the Pathways to Equitable Healthy Cities project (https://equitablehealthy-cities.org).

## 2 Study terminology

The term “slum” has been criticized by some as marginalising people living in areas labeled as slums, since they can face stigmatization and forced eviction (Dovey et al., 2020; Friesen et al., 2020; Thomson et al., 2020). Classification of deprived neighborhoods as a slum versus not a slum is also influenced by specific political and social histories of those places (Mayne, 2017). In discussing outputs of this study, we generally use the term ‘deprived area’ to describe places defined by slum conditions, informal settlements, or inadequate living or environmental conditions, as recommended by a recent review (Thomson et al., 2020). In instances where we refer to slums, as labeled by past studies or in government documents, we describe deprived areas that could benefit from resource allocation and improved infrastructure, but are not referring to the characteristics of people who live in them (Ezeh et al., 2017).

## 3 Study region

The GAMA region is the administrative and economic capital of Ghana, comprised of 12 districts that are subdivided into 406 neighbourhoods and 5,019 enumeration areas (EAs) in the 2010 census. It is the country’s most densely populated region (pop: ~4 million living within 1,507.5 km^2^ in 2010). At GAMA’s urban core is the Accra Metropolitan Area (AMA; ~225 km^2^), an area of concentrated economic activity with numerous opportunities for migrants in Ghana, and thus has been the geographic focus of previous slum identification studies in Accra (Accra Metropolitan Assembly, 2011; Engstrom et al., 2015). While AMA absorbed much of GAMA’s population growth before 2000, more recently districts outside of AMA have seen higher rates of growth, including the suburban residential area of Dome and the industrial areas of Tema and Ashaiman (Addae & Oppelt, 2019; Oduro et al., 2015). Though considered rural just several decades ago, these areas are now well incorporated into the greater Accra urban system and include satellite hubs of urban and economic activities. In 2010, these suburban areas already accounted for nearly half of GAMA residents, and their populations have steadily grown (Addae & Oppelt, 2019).

Most GAMA residents lived in EAs classified as urban by the 2010 census, with only a small fraction (8%) of residents living in areas classified as rural, which we label as peri-urban in the figures of this paper. Ghana does not have an official definition that distinguishes peri-urban from rural EAs; however, we used the term peri-urban to describe ‘rural’ designated EAs that are located within the administrative border of GAMA.

## 4 Methods

### 4.1 Data sources

#### 4.1.1 Slum classification for the Accra Metropolitan area (AMA)

We obtained a spatial map of 78 slum pockets and settlements for the AMA from a detailed field mapping exercise that was jointly conducted in 2011 by the Accra Metropolitan Assembly and UNHabitat, and produced a slum/non-slum dichotomy map (referred to here as the AMAUH map) (Fig. A1). Detailed information about the mapping outputs are described elsewhere (Accra Metropolitan Assembly, 2011). Briefly, the exercise identified slums using a combination of aerial photography, household income data from the municipal income classification scheme, data on population density and housing variables from the 2000 census, and interviews conducted with slum dwellers and members of the city assembly (Accra Metropolitan Assembly, 2011). The AMAUH outputs were spatially harmonized with shapefiles of EAs from the 2010 census by Engstrom et al. (2017). Enumeration areas are the smallest administrative geographic units in Ghana, with a median population of 689 [interquartile range (IQR): 486–940], and an average size of 0.05 km^2^ in urban GAMA.

#### 4.1.2 Housing, population density, and environmental quality predictor variables for GAMA

We collated spatial data from publicly-available administrative and remote sensing databases that reflected household and environmental characteristics for all of GAMA (Engstrom et al., 2017, 2015; UN-Habitat, 2016) (details in Tables A1 and A2). We selected these variables based on the identified housing and environmental attributes of slums in previous studies in Accra (Engstrom et al., 2017, 2015; Jankowska et al., 2011; Owusu et al., 2021; UN-Habitat, 2016) and then iteratively finalized our variable list based on input from Ghanaian-based researchers with expertise in urban poverty and development in Accra. Geocoded EA boundaries according to the 2010 Ghana geography were obtained from the Geographic Information System (GIS) team of the Ghana Statistical Service which we linked to the population data of the census to calculate the population density of each EA.

Spatially-resolved data on housing characteristics were obtained from a 10% random sample of the 2010 Population and Housing Census. Household data were extracted from census questions related to the UN-Habitat’s slum criteria which included: drinking water source; type of sanitation facility; ownership of dwelling; type of dwelling; housing materials used for the roof, floor, and walls; household size; and number of bedrooms. House-hold size was divided by the number of bedrooms to estimate crowding. Data on cooking fuel type and form of rubbish disposal were also extracted to assess physical hazard vulnerability (Jankowska et al., 2011). We combined some categorical variables based on the distribution of census categories and the aggregation of improved versus unimproved categories defined by UN-Habitat (Engstrom et al., 2017; UN-Habitat, 2016) (Table A1). Each categorical variable was expressed as a percentage of households in each EA.

We included elevation as an indicator of risk of flooding using Digital Elevation Model (DEM) data from the National Aeronautics and Space Administration (NASA) (https://lpdaac.usgs.gov/products/ast14demv003/) with 30 m resolution. Zonal statistics were used to estimate the mean elevation for each EA and its 5-km buffer. The elevation of the 5-km buffer was subtracted from the EA-level elevation mean to calculate the difference in elevation for each EA relative to the surrounding area.

Environmental quality via greenness was assessed using the normalized difference vegetation index (NDVI) measure, which quantified the abundance of vegetation. Landsat-8 satellite images were obtained from the United States Geological Survey (USGS) EarthExplorer for southern Ghana (U.S. Geological Survey, 2015). We selected satellite images taken between December 6, 2015 and January 7, 2016 which were the first available images closest to the census year (2010) that also had minimal cloud cover (<10%). Landsat-7 data prior to 2013 were unavailable due to satellite scanline errors. Zonal statistics were used to calculate means of the 30 m-resolution NDVI scores for each EA.

### 4.2 Statistical analysis

#### 4.2.1 Fitting the slum model

We first fitted a series of Bayesian regression models with AMUAH slum classification as a function of 18 independent variables that encompassed housing features, population density, and environmental quality in AMA. A complete list of independent variables and their definitions is provided in Tables A1 and A2.

Our dependent variable (*Y_i_*) was categorized as 1 if the EA was classified as a slum in the AMAUH field mapping study or 0 otherwise, and *X_i_* was a vector of 18 EA-level predictor variables. We assumed that *Y_i_* followed a Bernoulli distribution with parameter *p_i_* representing the probability of the i^th^ EA being a deprived ‘slum’ area.

Model 1: *Y_i_* ~ *Bernoulli*(*p_i_*)

$$logit\left(p_{i}\right)=\alpha +\beta X_{i}$$

where β is an 18-dimensional vector of coefficients capturing the linear relationship between each of the predictor variables and the logit of the probability of being a slum; and the constant α is the intercept which captures the overall probability when all predictor variables are simultaneously equal to zero. The coefficients *α* and β were assigned normal prior distributions with a mean of zero and standard deviation of 0.98. The specification β ~ N(0,0.98^2^) corresponded to a 95% odds ratios interval of exp(±1.96 × 0.98) which is equal to [0.15, 6.8], a reasonable range for Bayesian generalized linear models with a binary outcome (Wakefield, 2013).

In a second regression model, we included neighbourhood-level random effects to evaluate whether this improved predictive performance.

Model 2: *Y_ij_* ~ *Bernoulli*(*p_ij_*)

$$logit\left(p_{ij}\right)=\alpha +\beta X_{ij}+V_{j}$$

In Model 2 the fitted model also assumed a Bernoulli distribution for the outcome with parameter *p_ij_* representing the probability of being a deprived ‘slum’ area for the *i*^th^ EA in the *j*^th^ neighbourhood; and *V_j_* is the EA random effect at the neighbourhood level. In this model, *V_j_* is an unstructured EA random effect, where the model is fitted to the data assuming that the neighbourhood random effects were independent, *a priori*. The prior for the *V_j_* follows independent zero mean normal distributions, with a weakly-informative half-Cauchy prior for the standard deviation (Gelman, 2006).

For all regression models, we used Markov chain Monte Carlo (MCMC) methods to sample from the posterior distribution with 20,000 post burn-in iterations. The fitted models were run using NIMBLE, a hierarchical statistical modeling package in R (de Valpine et al., 2017). We configured an automated factor slice sampler for *α* and β, which samples more efficiently than alternative MCMC algorithms (Tibbits et al., 2014). Trace plots were visually evaluated for model convergence.

Potential multi-collinearity between predictor variables was assessed through visual inspection of a correlation matrix and a chi-square test for independence between binary predictors. As some pairs of predictor variables had a moderate to high correlation (Pearson r = 0.7–0.9) (Fig. A2), we also fitted Bayesian versions of lasso (Biswas & Lin, 2012) and ridge regression (Reich & Ghosh, 2019) models. The former assumed a double exponential prior for the predictor coefficients and the latter assumed a zero mean normal prior with the precision following a flat inverse gamma prior distribution. The resulting posterior distributions of the predictor coefficients were nearly identical under the different prior specifications (Fig. A3). Our final model is based on the zero-mean normal prior distribution with a standard deviation of 0.98 for predictors, which is the original specification recommended for Bayesian generalized linear models with a binary outcome (Wakefield, 2013).

Our model outputs were the posterior distribution of the probabilities of EAs being deprived ‘slum’ areas and the mean summary of the posterior distribution of the model coefficients, which can be interpreted as odds ratios (OR) and 95% credible intervals (CrInt) (Szumilas, 2010).

#### 4.2.2 Model selection

We undertook a model selection process to identify a parsimonious and generalizable model with the best predictive performance and extended the deprived ‘slum’ area prediction to all of GAMA. We evaluated the fit of models 1 and 2 with crossvalidation of 2.5%, 3.7%, 5.0%, 6.2%, 7.5%, 8.7%, and 10% random samples of EAs in AMA. We excluded these random samples from the generative models prior to fitting, and then predicted deprived area probability estimates of the excluded EAs for each respective model. We assessed the posterior distributions of the predicted deprived area probabilities and compared them against their fitted probability values. In each cross-validation comparison, the mean square error (MSE) values were assessed as indicators of predictive performance. We then evaluated the model assumptions using diagnostic plots of the predictive posterior distributions. The model fit was also evaluated using the Watanabe-Akaike Information Criterion (WAIC) which is the generalized version of the Akaike Information Criterion that estimates prediction error while considering model simplicity to prevent overfitting (Evans, 2019; Gelman et al., 2014).

#### 4.2.3 Predicted probability of deprived EAs in the GAMA

After selecting a final model for prediction, we generated the predictions of EA-level deprived area probabilities for all urban EAs in GAMA. The predictions were restricted to EAs categorized as urban by the census, thus capturing areas that house 92% of GAMA residents. We did not extend our prediction to EAs classified as rural in the census because these peri-urban regions have different characteristics and contexts than the urban EAs included in our training model for central Accra. We spatially overlayed the predicted probabilities onto a map of GAMA, and summarized the percentage of urban population living in EAs falling into quintiles of deprived area probabilities (0–20%, 20–40%, 40–60%, 60–80%, and 80–100%). For clusters of three or more EAs identified as having a high deprived area probability (i.e., ≥0.80), we used Google Earth Pro version 7.3.3.7786 to retrieve satellite images from 2010, the same year as the census data, to qualitatively describe key environmental characteristics of these areas, including presence of greenspaces, road networks, building density, and nearby services.

#### 4.2.4 Local indicators of spatial association (LISA) analysis

We used local indicators of spatial association (LISA) analysis to identify clusters of housing and environmental attributes, and spatially compared these clusters with predicted deprived areas in GAMA (Anselin & Rey, 2010). We identified clusters of high or low values for each of the predictor variables across GAMA, and compared the maps of LISA clusters and EAs with high probability of being a deprived area.

The statistical analysis and mapping in this study were conducted in RStudio version 1.2.5042. The raster data extraction and preparation of density and environmental quality predictor variables were done in QGIS version 3.16.0.

## 5 Results and discussion

### 5.1 Model performance

We chose Model 1 (without neighborhood random effects) for prediction of deprived areas for all of GAMA because it had consistently lower MSE values than Model 2 and more accurately predicted deprived area classification of excluded EAs during cross validation (Table A3). Model 1 also had more precise 95% posterior credible intervals in the predictive posterior distributions compared with Model 2 (Figs. A4–A5). Model 2 had the smallest WAIC score, indicating best fit, but worse predictive performance.

### 5.2 Associations of predictor variables with AMAUH slum classification

**Fig. 1:**
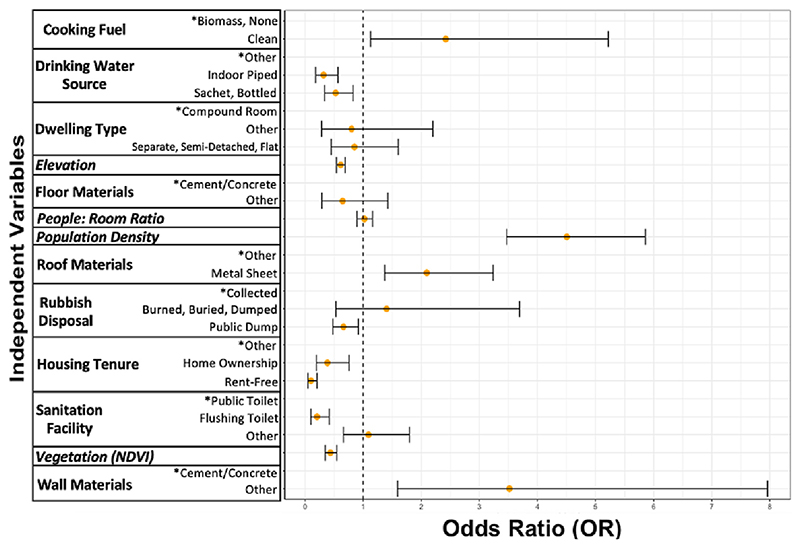
Associations of AMAUH slum classification with housing, population density, and environmental predictor variables for enumeration areas in the Accra Metropolitan Area^a^. Posterior summary of the odds ratios (orange circles) and their 95% posterior credible intervals. Reference variables are signified by an asterisk (*). Categorical independent variables are calculated as the percentage of households in an EA (range from 0 to 1). Italicized variables are continuous. ^a^Independent variable definitions and classifications are provided in Tables A1 and A2. (For interpretation of the references to colour in this figure legend, the reader is referred to the web version of this article.)

Variables associated with higher odds of an EA being classified as a deprived ‘slum’ EA in our fitted regression model included: greater population density; and a higher proportion of households with less durable wall materials, using clean cooking fuel sources like gas or electricity, and having metal sheet roofs (Fig. 1). Variables associated with lower odds of being classified as a deprived EA included: greater vegetation, higher elevation, and a higher proportion of households with improved drinking water sources (e.g., indoor piped or sachet and bottled water) compared to unimproved water sources (e.g., public taps or outdoor piped water), flushing toilets compared to public toilets, using a public dump for rubbish disposal compared to having rubbish collected, and rent-free tenure or home ownership compared to renting, perching, or squatting. The addition of independent neighbourhood-level random effects into the model resulted in similar outputs (Fig. A6).

### 5.3 Comparison of our prediction results with the AMAUH slum map

**Fig. 2:**
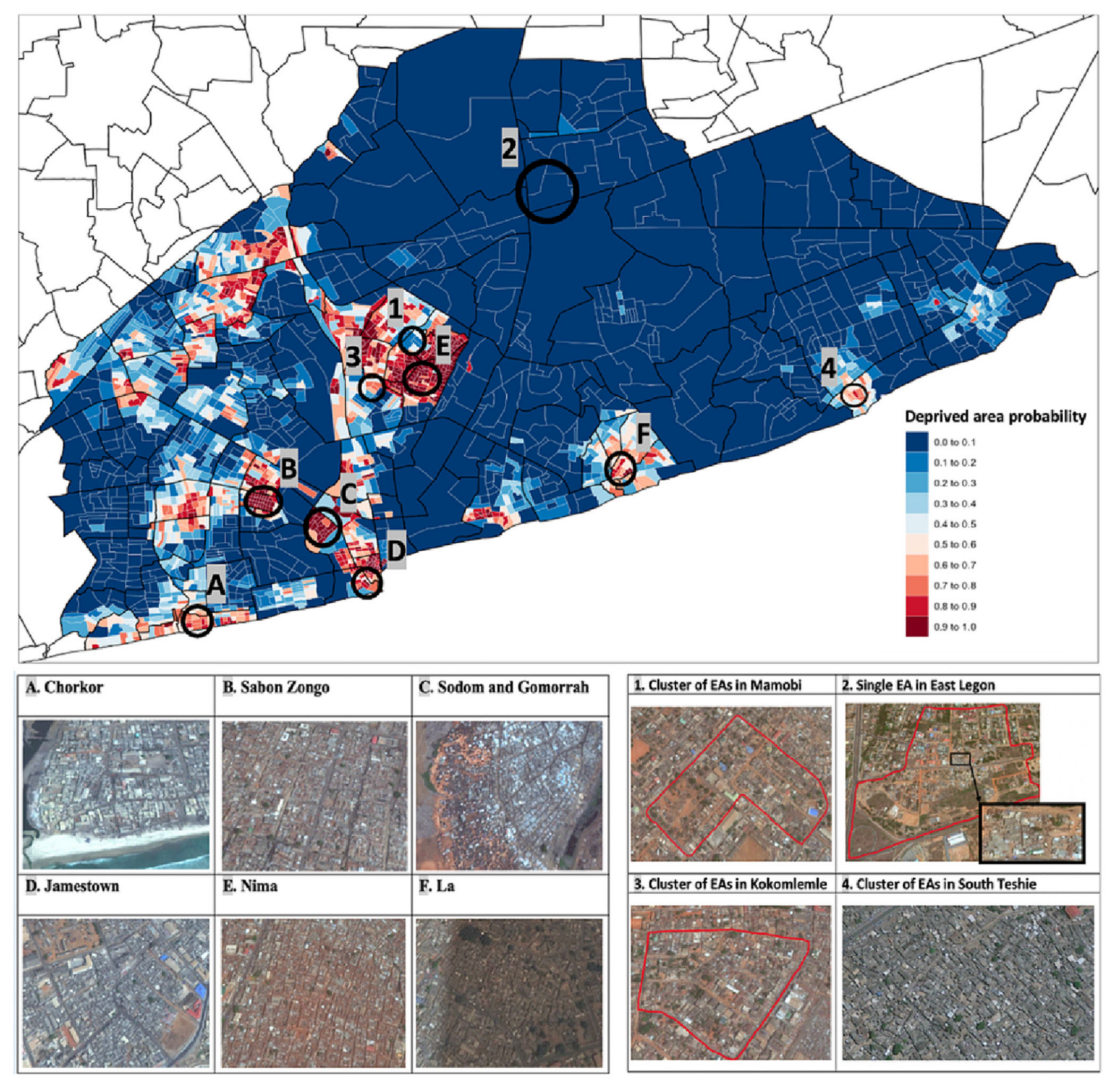
Predicted probabilities of being a deprived ‘slum’ area for enumeration areas (EAs) in the Accra Metropolitan Area (AMA). Areas labeled A-F signify established slum neighbourhoods that have clusters of high predicted deprived area probabilities in our model and were also classified as slums in the AMAUH map. Areas labeled 1–4 are where predicted deprived area probabilities in our model contrasted with the AMAUH map. EAs are shown in white boundary lines, while neighbourhoods are shown in black boundary lines. Maps Data: Google, ©2009-2021 CNES/ Astrium, Maxar Technologies.

In our model for AMA, 476 EAs (~20% of AMA EAs) had a high predicted probability of being a deprived area (≥0.80), 438 of which were also identified as slums in the AMAUH field mapping study. The locations of the deprived areas in our model (Fig. 2) were similar to the AMAUH map, and both identified EAs in well-established slums including Chorkor, Sabon Zongo, Sodom and Gomorrah, Jamestown, Nima and La neighbourhoods as having a high probability of being deprived (Fig. A1, Fig. 2). There were several notable discrepancies between our results and AMAUH’s, including 49 EAs with low deprived area probabilities (<0.20) in our model that were classified as slums by AMAUH. Several of these EAs were located within large and well-established slums including Mamobi, Sabon Zongo, and Chorkor (Accra Metropolitan Assembly, 2011; Jankowska et al., 2011). It is possible that our model identified more serviced and less deprived EAs within larger slums. For example, satellite imagery from 2010 shows that a cluster of such EAs in Mamobi had individual housing units surrounded by greenspace and other urban services including a police station, churches, and retail shops, indicating that these EAs may be higher-resourced and better serviced than the neighbouring EAs in this large slum area.

Emerging and temporary slums and other deprived areas are challenging to identify due to their small size and potentially fleeting presence, especially since identification methods for large geographic areas usually rely on data collected at a single time point and at higher spatial resolution. Our model may be less able to identify temporary or emerging deprived areas than field studies, and may miss smaller deprived areas that are nested within wealthier EAs where the features of high-income homes mask the vulnerabilities of a small group of households. For example, AMAUH identified a slum pocket within an EA in the high-income and green neighbourhood of East Legon, but the entire EA had a low probability (<0.20) of being a deprived area in our model. Small slum pockets rapidly emerge and are dismantled in East Legon as immigrants and squatters move into undeveloped plots of land on its periphery (Mireku et al., 2021). Satellite images of East Legon show a small cluster of densely-packed and informal households in 2010 that disappeared by 2016, when a new cluster of informal households appeared in a different location (Fig. A7). Still, a notable advantage of our approach is that it can be conducted as a first-step assessment and in contexts where field-based methods would be too expensive or otherwise infeasible. Our approach identified higher resourced EAs within low-income neighbourhoods such as in Mamobi, and our model can be applied to larger geographical areas at a lower cost than field-based mapping.

Our model identified 38 EAs with high probabilities of being deprived (≥0.80) that were not identified as slums in the AMAUH map. For example, 14 EAs in South Teshie had a high or moderate probability (≥0.60) of being deprived in our model but were not labeled as slums by AMAUH. Teshie is a mixed-income neighbourhood with clusters of poor households along the southern coast (Farvacque-Vitkovic et al., 2008; Fiasorgbor, 2013). We identified deprived areas in Teshie that are not locally recognized as “slums” but share many of the same living and environmental conditions as established slums. Thus our modeling approach may identify areas that would benefit from infrastructure development and increased allocation of resources but are less visible to decision-makers because they are located outside of established slums.

Although our studies had similar spatial patterns, we classified fewer EAs in AMA as having a high probability (≥0.80) of being a deprived area than a recent study that identified 809 EAs as slums using a machine-learning approach for the AMA (Engstrom et al., 2017). This discrepancy may be because Engstrom and colleagues trained their model using EAs at the extremes (i.e., either established slums or very wealthy neighbourhoods), which could classify low- or lower middle-income EAs as having a high slum index since their characteristics differ from the wealthiest neighbourhoods. We also used a conservative cut-off of 0.80 for labeling areas as “deprived” and shifting to a lower cut-off (≥ 0.50) increased our number of deprived EAs (n = 958).

#### 5.3.1 Spatial patterns of deprived ‘slum’ areas in GAMA

**Fig. 3:**
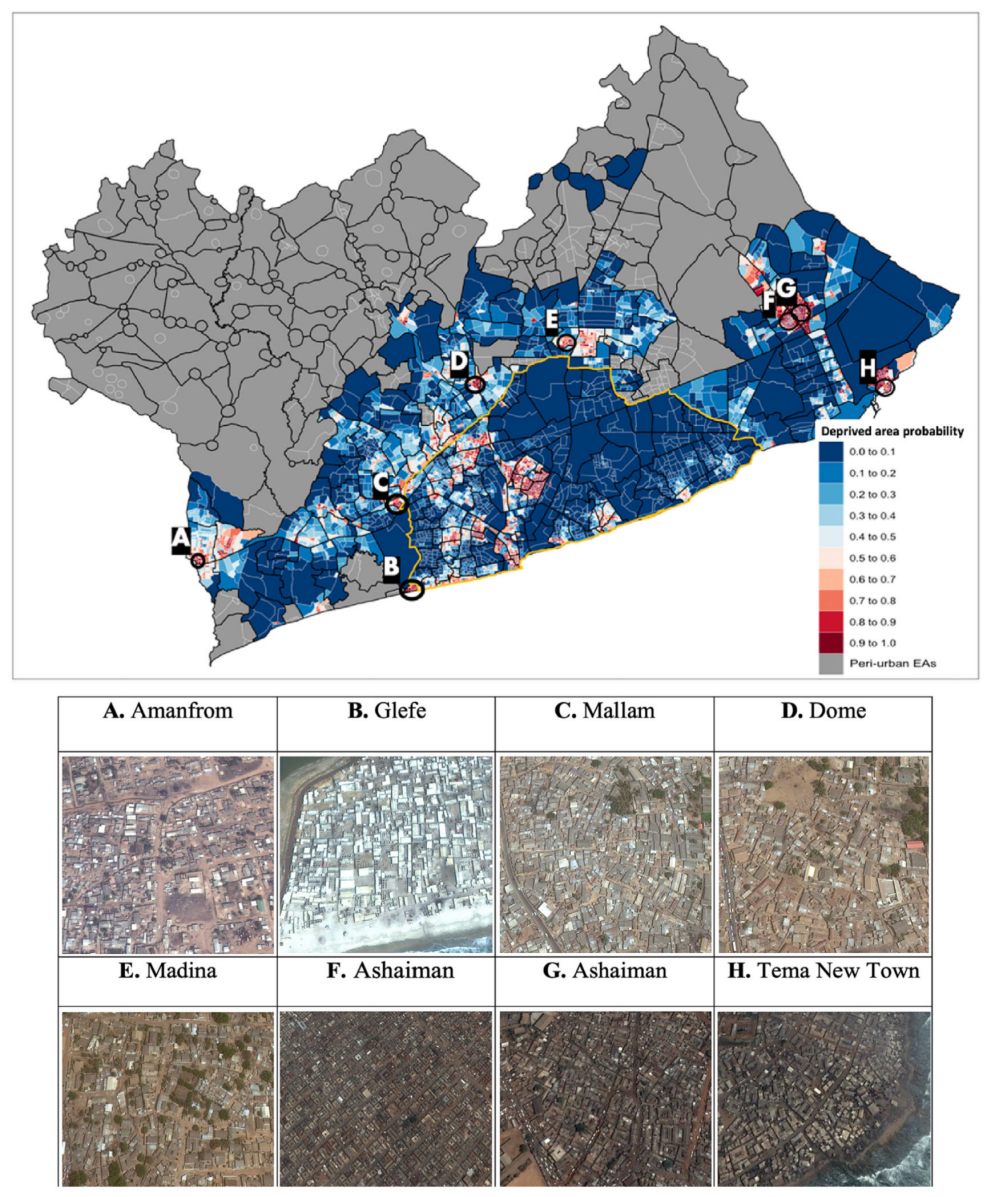
Posterior predictive probabilities of being deprived areas based on a logistic regression model for urban enumeration areas (EAs) in the Greater Accra Metropolitan Area (GAMA). Areas on the map identified with letters are examples of clusters of at least 3 EAs outside the urban core (Accra Metropolitan Area, outlined in yellow) with a high probability (≥0.80) of being deprived in our model. The corresponding satellite images for these areas are shown below. EAs are outlined with white boundary lines, and neighbourhoods outlined with black lines. Maps Data: Google, ©2009-2021 CNES/ Astrium, Maxar Technologies. (For interpretation of the references to colour in this figure legend, the reader is referred to the web version of this article.)

Our predictive model identified 899 urban EAs (19% of all urban EAs) in GAMA with a high predicted probability (≥0.80) of being deprived, nearly half of which were located in EAs outside of AMA (n = 423). These EAs were geographically dispersed across most of GAMA except for peri-urban areas in the north which are administratively considered ‘rural’ in the census and thus excluded from prediction. Based on population data from the 2010 census, we estimated that approximately one in five residents (745,714 people) in urban GAMA lived in EAs with a high predicted probability of being deprived, and over a third of residents (1,233,451 people) lived in an EA with a moderate to high probability (≥0.60) of being deprived (Table 1; Fig. A8). We found large clusters of deprived EAs within inland urban areas (e.g., Amanfrom, Mallam, Dome, Madina, and Ashaiman) and along the southern coast (e.g., Glefe and Tema New Town) (Fig. 3). Satellite imagery of these clusters generally showed densely packed buildings connected mainly by footpaths and with limited access to roads.

The Madina neighbourhood (Fig. 3E) in northern AMA had a high deprived area probability in our model and is a low-income community that emerged from a forced resettlement program of Nima/Mamobi residents to make space for a highway project in the 1970s (Nyametso, 2012). Displaced residents from forced evictions may not be re-settled into neighbourhoods with better living conditions. Another deprived cluster identified in our model was in Glefe (Fig. 3B), a densely populated and largely unplanned neighbourhood that is susceptible to flooding and erosion because of its close proximity to Lake Bebu (Amoako, 2018; Amoako & Inkoom, 2018; Amoani et al., 2012; Owusu et al., 2019). Glefe also lacks access to roads, and adequate water and sanitation, which poses further health risks to its residents, and by all descriptions is a deprived neighbourhood (Owusu et al., 2019). A separate cluster of identified deprived EAs along the coast in Tema New Town (Fig. 3H) was developed without compliance with formal planning regulations and lacks proper drainage systems which, similar to Glefe, places its residents at high risk of exposure to floods (Gyan, 2019).

When comparing our results with satellite images, we observed a diversity of urban planning in Ashaiman (Fig. 3F–G), a large and long-established slum in Tema (Abass & Kucukmehmetoglu, 2021) with 199 EAs having a high probability of being deprived (≥0.80) in our model. The area is generally characterized by high density housing and limited greenspace. Eastern Ashaiman (Fig. 3G) is more unplanned with limited access to road networks whereas western Ashaiman EAs (Fig. 3F) are more planned (i.e., structured blocks of buildings) with direct access to roadways. Ashaiman was a target area for slum upgrading programs conducted by the Tema Ashaiman Municipal Slum Upgrading Facility (TAMSUF) in 2009 (Danso-Wiredu & Midheme, 2017), so it is possible that some of these EAs were undergoing re-development during the 2010 census.

#### 5.3.2 Spatial patterns of housing, density, and environmental characteristics in the GAMA

**Fig. 4:**
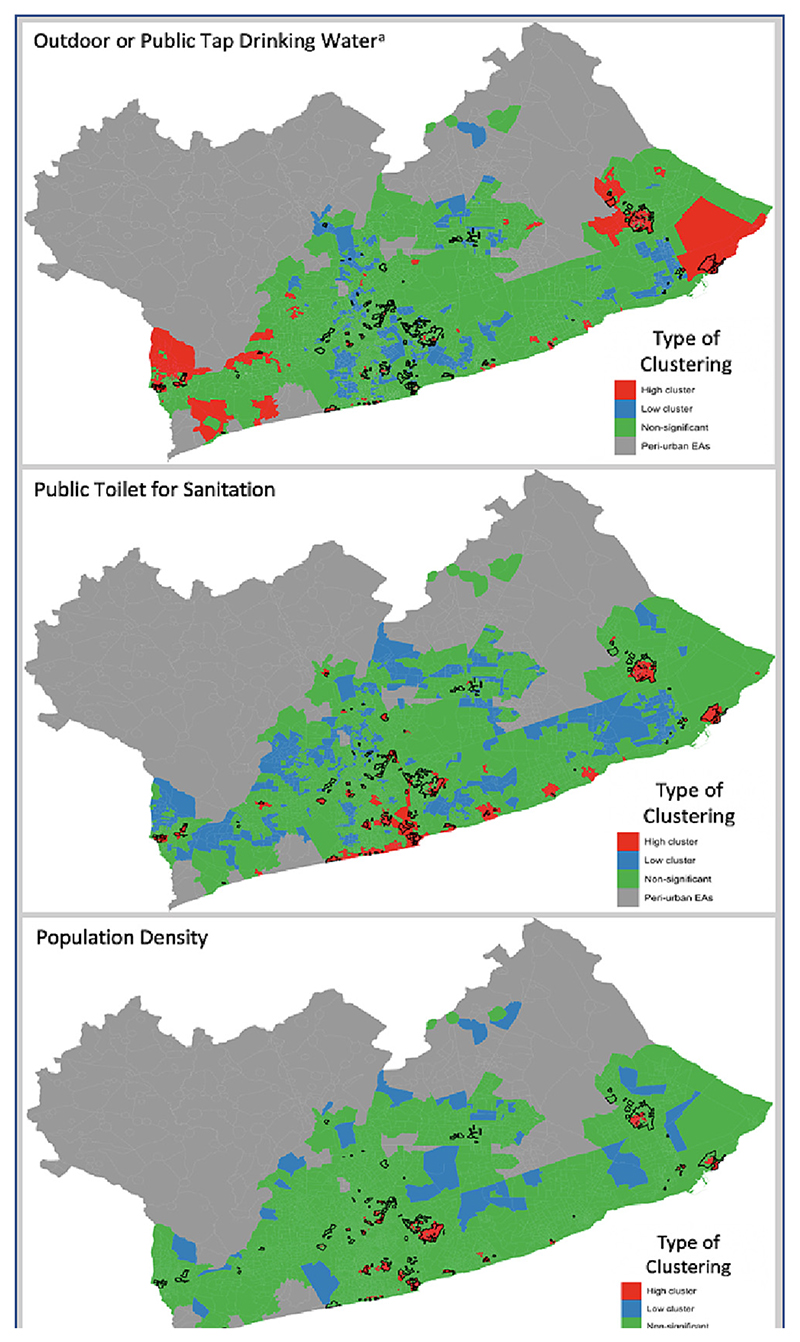
Spatial clustering of housing, population density, and environmental quality attributes in the Greater Accra Metropolitan Area (GAMA). Results from a LISA spatial autocorrelation test. Each attribute was calculated at the EA-level, and EAs with a high probability of being deprived (>80%) are outlined in black. Red shading indicates statistically significant clusters of high values (p < 0.05), blue shading indicates statistically significant clusters of low values (p < 0.05), and green shading indicates no observed spatial correlation (p > 0.05). Each of the independent variables demonstrated significant clustering (p < 0.05). Population density is a continuous variable, whereas the other attributes were measured as the percent of households within an EA (range 0–1). The outdoor tap or public tap variable (^a^) also included other less commonly observed drinking water sources other than indoor piped water or sachet/bottled water. (For interpretation of the references to colour in this figure legend, the reader is referred to the web version of this article.)

We observed local clustering of high values for households using public toilets [Global Moran’s I (GMI) = 0.63, p < 0.001] in low-income neighbourhoods such as Nima, Jamestown, Sodom and Gomorrah, South Teshie, Ashaiman, and Glefe (Fig. 4). Population density had high value clusters (GMI = 0.65, p < 0.001) in well-established slum neighbourhoods including Sabon Zongo and Nima. Outdoor drinking water sources (e.g., public taps) had high clusters in deprived areas such as Sodom and Gomorrah, Tema New Town, and Ashaiman (GMI = 0.36, p < 0.001).

Deprived area identification alongside information on clusters of housing and environmental attributes can guide policies and intervention programs, including multiple-objective upgrading programs. We observed clusters of high population density in many deprived areas in central Accra, where targeted housing development programs combined with the enforcement of building codes could help to relieve congestion and overcrowding while also increasing the stock of safe, durable housing units in the city center. Lower housing costs and improved access to essential services to neighbourhoods along the urban periphery of GAMA could reduce influx of migrants into crowded parts of the city (Ren et al., 2020). Several large deprived neighbourhoods including Ashaiman had significantly high clustering of homes relying on public toilets and outdoor drinking water sources. These areas could be prioritized for infrastructure upgrading in the longer term (Appiah-Effah et al., 2019), and as a near term solution, for public health education programs focused on hygiene and water disinfection at the household-level (Machdar et al., 2013).

Our study has a number of strengths that build on previous slum identification work in Accra and other cities. Our study extends deprived area identification to the suburban areas outside of Accra’s urban core, which are the fastest growing regions in GAMA. Our integration of mixed data sources, including publicly-available remote sensing and housing quality data from the census, enabled us to measure a range of land-use (neighbourhood) and indoor (housing) features and access to services that all contribute to an area being deprived. Census, survey, and remote-sensing data are repeatedly collected for Ghana and other low- and middleincome countries, and our data and scalable approach could be adapted for future studies that assess changes in the spatial–temporal patterns and characteristics of existing deprived areas and identify new deprived areas that emerge as cities develop and change over time. Finally, our novel use of a Bayesian spatial modeling approach provides a flexible and powerful alternative to the machine-learning approaches used in previous slum identification studies in Accra and elsewhere.

Our study also has several limitations to consider for future research. First, there are other social and environmental variables that might allow for better prediction of deprived areas but were not available at the same time as the census (e.g., air pollution, noise, or income levels). Second, we were unable to compare our predicted results for all of GAMA against field mapping results or another gold standard, though we did find consistency between our results and descriptions of deprived areas in GAMA in past literature, satellite imagery of these areas, and iterative discussions with local researchers with expertise in urban poverty and development. Third, our study used the most recently available census data from 2010 which may not accurately reflect current urban development in Accra, as would be the case for many studies of rapidly developing and changing urban settings like Accra. When released, the 2021 census will provide a near-term research opportunity to efficiently adapt our analysis and assess changes over time, including the appearance of new deprived areas, and to evaluate Ghana’s progress towards meeting SDG-11. Fourth, we assume that our spatial predictors were representative of the time period when the AMAUH slum mapping exercise was conducted (2011). This is reasonable for the 2010 census data but may not hold for NDVI measures from 2016. It is possible that deprived and non-deprived areas had differential changes in vegetation between 2010 and 2016, though a study of changes in vegetation abundance in Accra found no significant changes in vegetation related to socioeconomic status between 2002 and 2010 (Stow et al., 2013). Finally, we cannot rule out the possibility of residual spatial confounding between the neighbourhood-level random and fixed effects in our models which would bias our model estimates (Reich et al., 2006), though the predictor variable coefficients were similar in models with and without independent effects, indicating that large spatial confounding is unlikely in our study.

## 6 Conclusions

Our study combined administrative and remote sensing data to identify deprived areas across the rapidly urbanizing GAMA. Nearly one in five urban EAs in the GAMA had a high predicted probability of being deprived in our study, accounting for an estimated 745,714 out of 3,433,979 residents in 2010. These EAs are dispersed across GAMA, however we identified several large clusters along the coast and in eastern GAMA. The identified deprived EAs were relatively heterogeneous in their housing and environmental conditions, but shared some common features including a higher population density, lower elevation and vegetation abundance, and less access to indoor piped water and sanitation.

Our study methods and outputs are timely, as they are synchronous with the ongoing global policy agenda to upgrade slums as a major goal of the SDG-11 to make cities inclusive, safe, resilient, and sustainable. Our spatially-resolved census and remote sensing data inputs are publicly available for many cities in sub-Saharan Africa and are collected over time, such that our modeling approach could be repeated in other urban contexts where data gaps on slums and other deprived areas exist. By enabling cities to produce accurate, repeatable, and scalable maps of their deprived areas, our study can help them to identify vulnerable populations, more equitably allocate housing and public services, prioritize areas for infrastructure development, and better protect their residents from natural disasters and disease.

## Supplementary Material

Appendix A

## Figures and Tables

**Table 1:** Number and percentage of urban EAs and their populations by quintile of the posterior probability of being deprived in the Greater Accra Metropolitan Area of Ghana.

Predicted Probability of Being Deprived	Number (%) of Urban EAs	Number (%) of Urban EA Residents
<0.20	1,642 (35.6%)	1,124,752 (32.8%)
≥0.20 & <0.40	840 (18.2%)	610,354 (17.8%)
≥0.40 & <0.60	626 (13.6%)	465,422 (13.6%)
≥0.60 & <0.80	608(13.2%)	487,737 (14.2%)
≥0.80	899 (19.5%)	745,714 (21.7%)
Total	4,615 (100%)	3,433,979 (100%)

## Data Availability

The deprived area probability estimates for this study will be made available at http://equitablehealthycities.org/data-download/. Data from a random 10% sample of households enumerated in the 2010 Population and Housing Census are publicly available and can be downloaded from the Ghana Statistical Services (GSS) online data catalogue (https://www2.statsghana.gov.gh/nada/index.php/catalog/51).
