# Supplementary material for: Identifying deprived “slum” neighbourhoods in the Greater Accra Metropolitan Area of Ghana using census and remote sensing data
Source: World Dev. Author manuscript; Available in PMC 2023 Sep 27. (PMC7615130; doi:10.1016/j.worlddev.2023.106253)
Supplement: Appendix A [file EMS188100-supplement-Appendix_A.docx]

# **Appendix: Supplementary Information**

**Supplementary Figures:**

Figure A1: Mapped classification of EAs as slum or non-slum based on whether the EA centroids fell within a slum settlement or pockets in the AMAUH slum map (UN-Habitat, 2011).

**
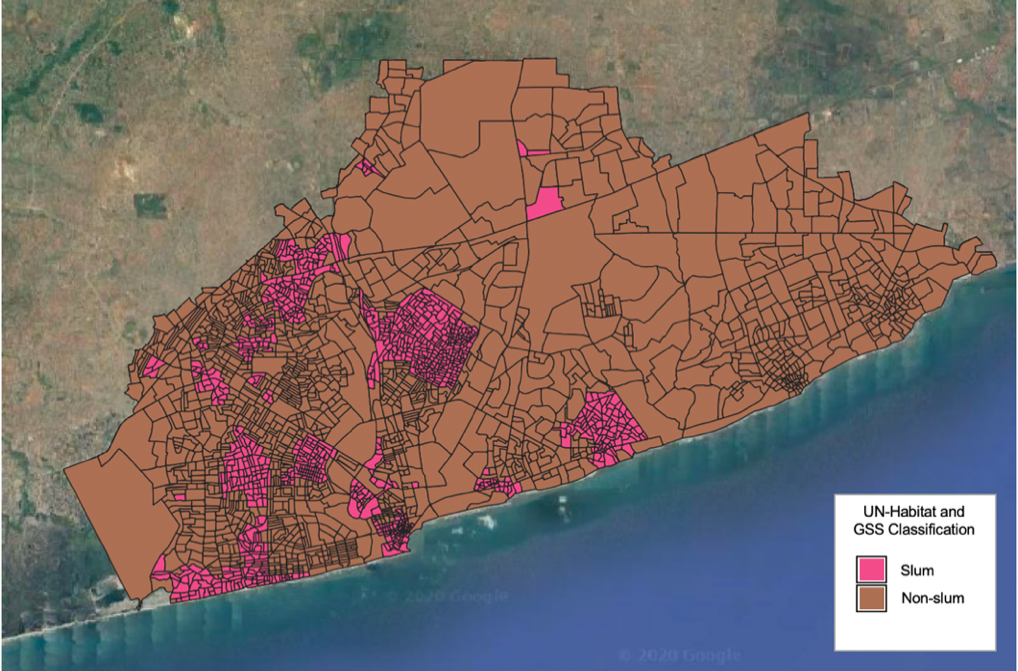
**

Figure A2: Pearson correlation coefficient matrix between the housing, population density, and environmental predictor variables (range from -1 to 1).


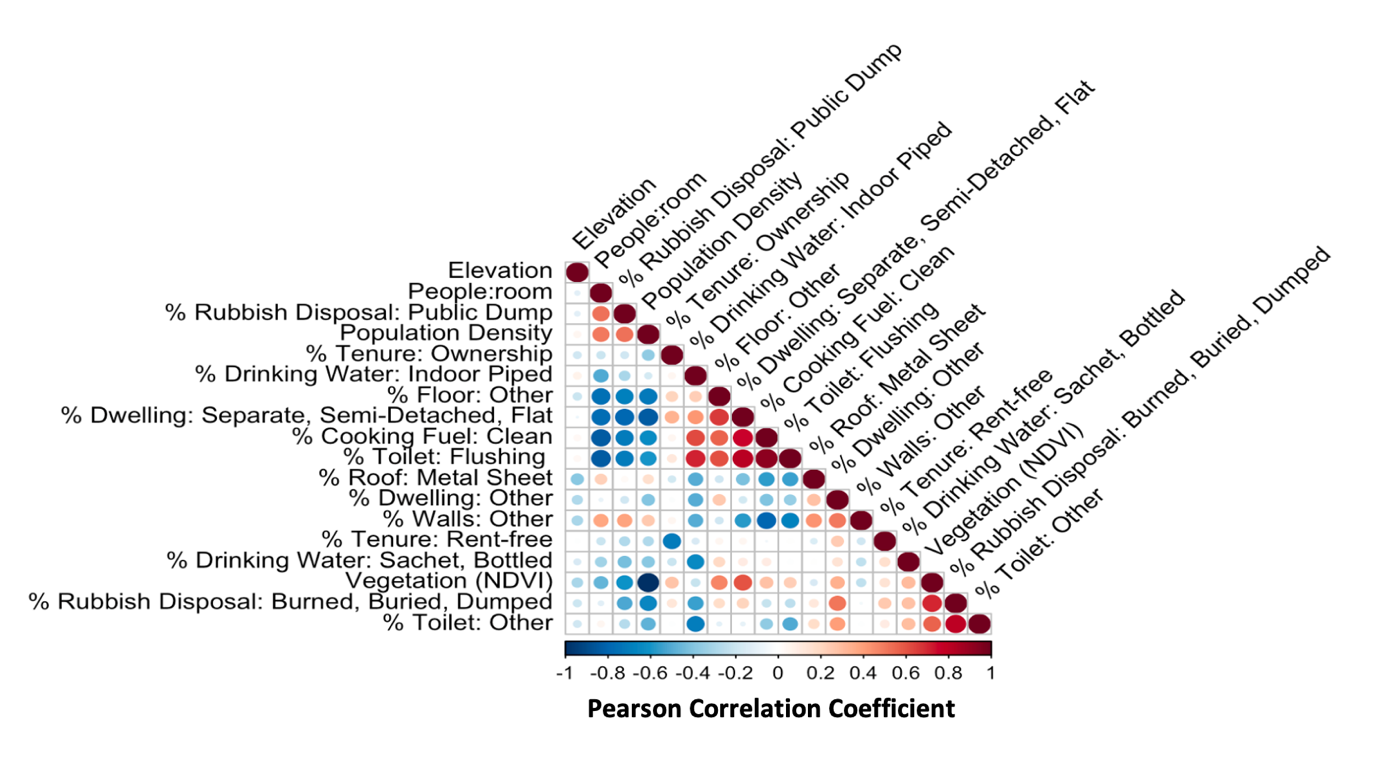


*Comparing models with and without independent neighbourhood-level random effects when measuring the association between predictor variables and probability of an EA being a deprived area*

There were three notable exceptions with reversed associational direction and significance including the proportion of homes using clean cooking fuel versus all other kinds of cooking fuel, proportion of homes with a metal sheet roof versus all other roofing materials, and proportion of homes using a public dump for rubbish disposal versus rubbish collection. As Model 2 had a better fit but was not selected for prediction due to poor predictive performance, it is possible that these significant associations in Model 1 were more appropriately captured by the neighbourhood random effects in Model 2.

Figure A3: Associations of deprived area classification with housing, population density, and environmental predictor variables for enumeration areas in the Accra Metropolitan Area. A comparison of posterior summary of the odds ratios (points) and 95% posterior credible intervals, using different prior specifications for the β coefficients.


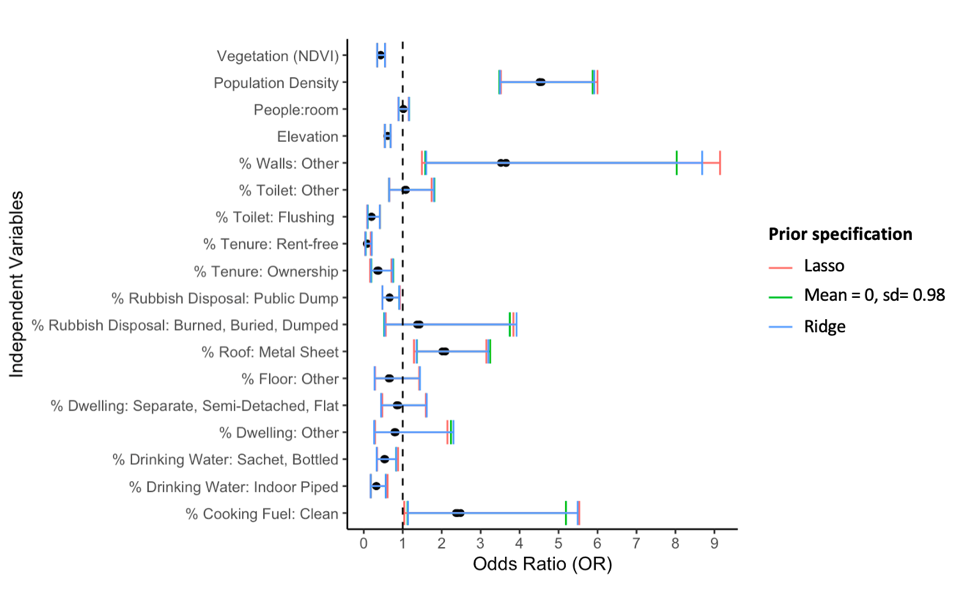


Figure A4: The posterior distribution of EA deprived area probability predictions in the AMA. These histograms show comparisons of four EAs (same between Model 1 and Model 2) that were excluded at random from the fitted model and subsequently predicted.


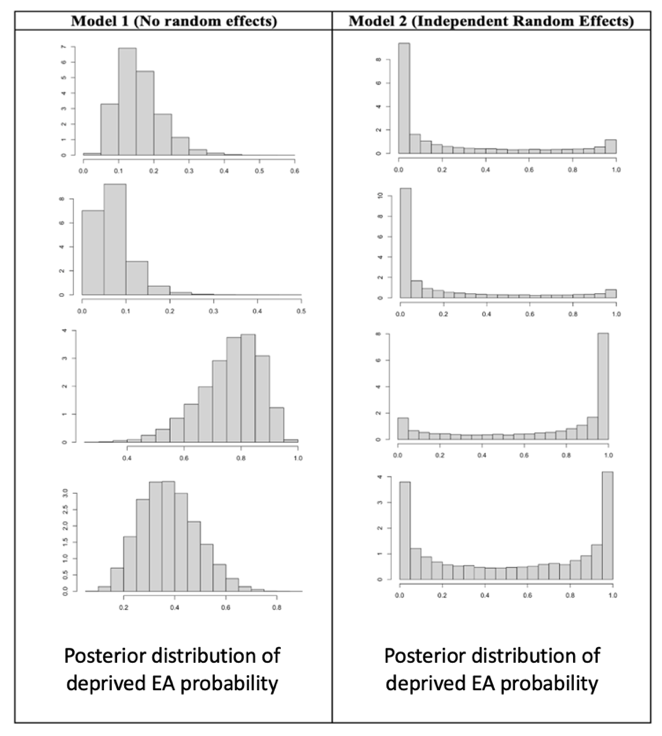


Figure A5: Segment plots of the posterior distribution of deprived area probability predictions in the AMA. The top segment plot (A) is showing the deprived area probability distributions from Model 1 with no independent random effects, whereas the bottom segment plot (B) is the deprived area probability distributions from Model 2 with independent neighbourhood random effects. Each segment plot is showing the point estimates and 95% posterior credible intervals of the deprived area probabilities for a sample of the same 100 EAs.


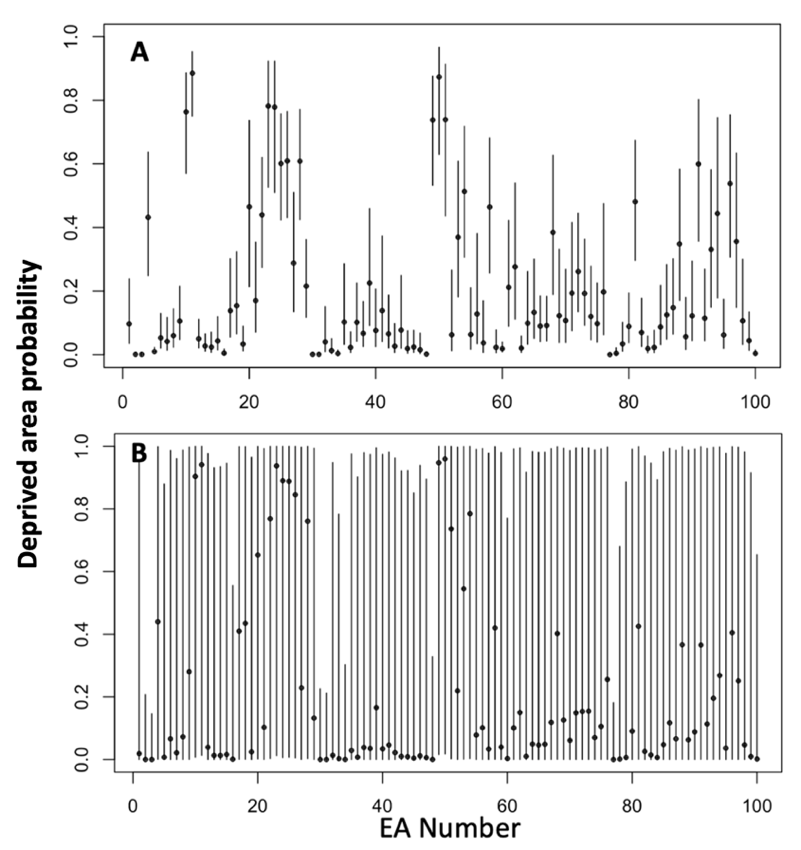


Figure A6: Associations of deprived area classification with housing, population density, and environmental predictor variables for enumeration areas in the Accra Metropolitan Area. A comparison of the Bayesian logistic regression models without independent random effects (Model 1) and with independent neighbourhood-level random effects (Model 2). Posterior summary of the odds ratios (black circles) and 95% posterior credible intervals are shown. Reference variables are signified by an asterisk (*). Categorical independent variables, indicated by variables with categories on the right side, are calculated as the percentage of households within an EA (range from 0-1). Italicized variables are continuous.


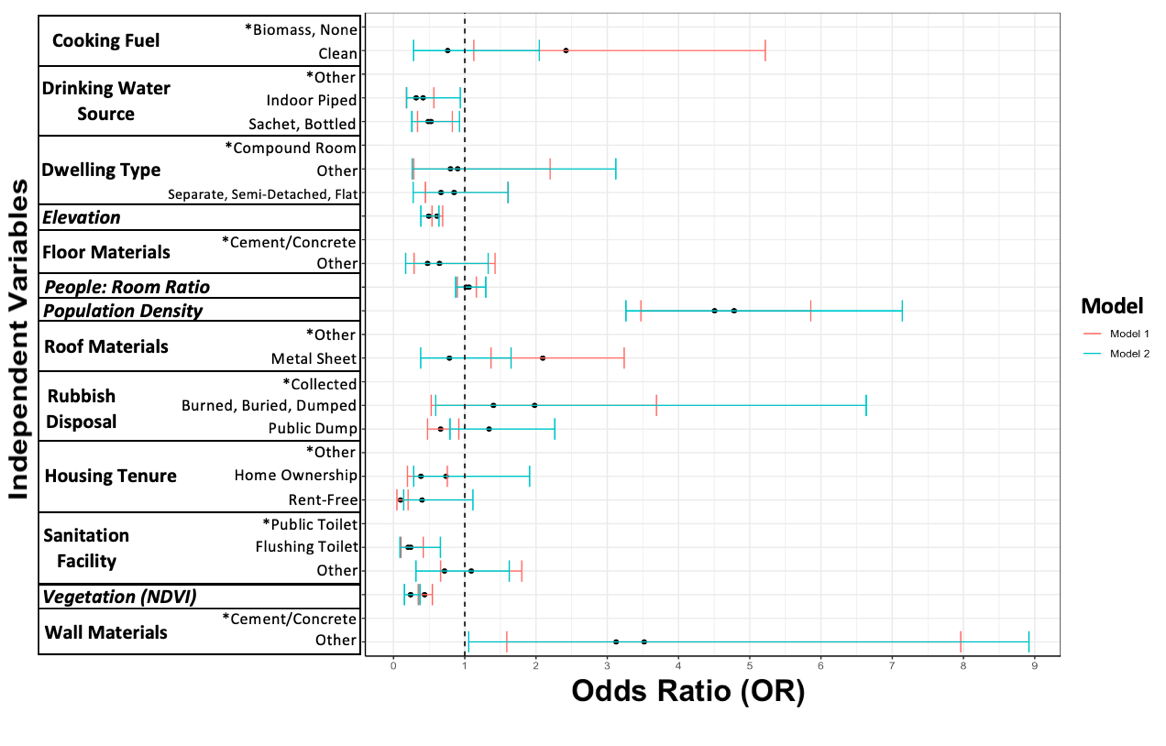


Figure A7: Temporary and emerging slums in East Legon, Accra between 2010 (A) and 2016 (B). In 2016 it can be observed that the slum pocket identified in image A was redeveloped, and a new slum pocket identified in image B emerged.


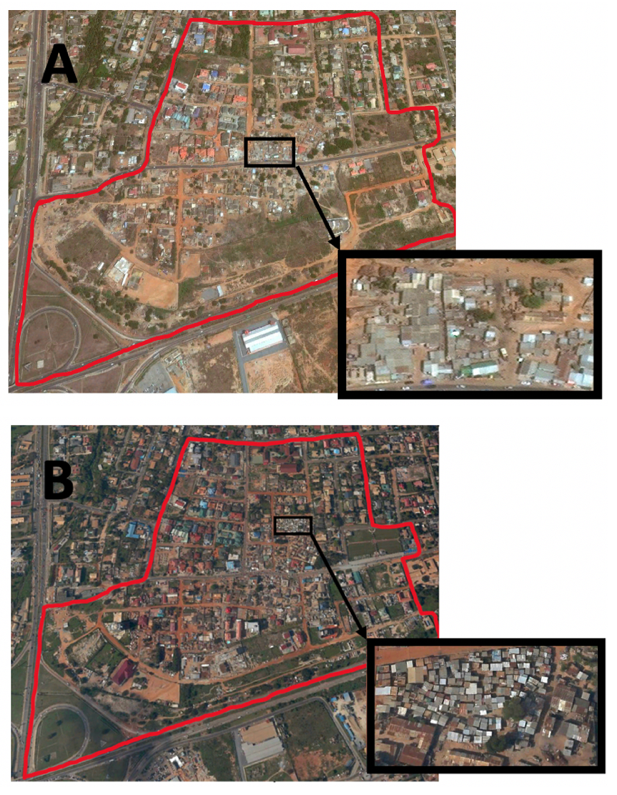


Figure A8: Histograms showing the distribution of deprived area probabilities in urban EAs within the GAMA, using A) 5 bins, B) 10 bins, C) 20 bins, and D) 40 bins.


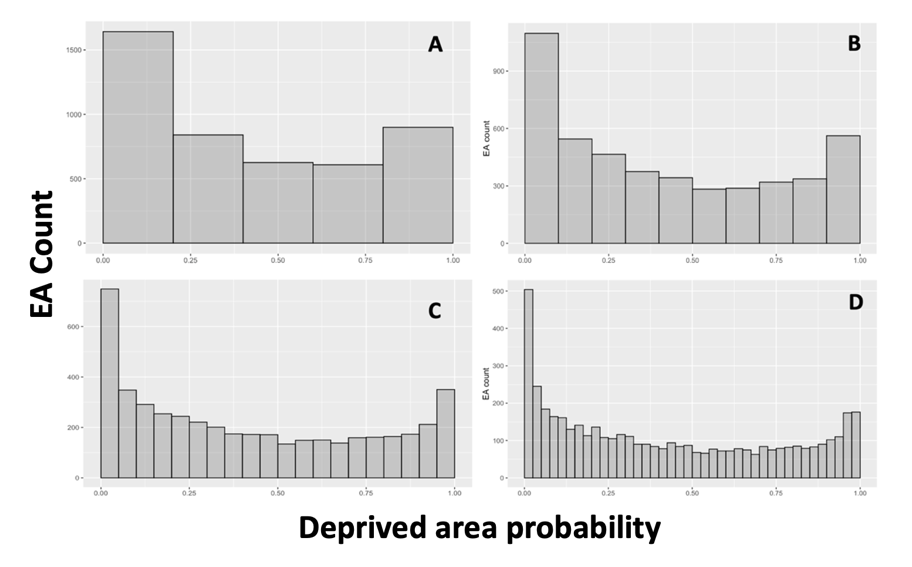


**Supplementary Tables:**

**Table A1.** Variables derived from categorical census questions at the EA-level in urban GAMA (2010 census). Bolded variable categories illustrate the reference categories in the regression models.

| **Indicator** | **Aggregated variable categories** | **Categories combined to create variable (% of GAMA households)** |
| --- | --- | --- |
| Household drinking water source | **“Other” drinking water source** | Outdoor piped: 29%  Public tap: 9.0%  Tanker: 3%  Borehole/Tube well: 1.0%  Protected Well or Spring: 0.7%  Rainwater: 0.1%  Unprotected Well or Spring: 0.1%  River/Stream/Dugout/Pond/Lake: 0.1%  Other: 0.1% |
|  | Indoor piped drinking water | Indoor Piped Water: 28.1% |
|  | Sachet or bottled drinking | Sachet Water: 28.2%  Bottled Water: 1.1% |
| Tenure type/form | **Renting, perching, squatting, or other tenure** | Renting: 49%  Perching/Squatting/Other: 1.9% |
|  | Owner-occupied tenure | Owner-occupied: 30.5% |
|  | Rent-free tenure | Rent-free: 18.3% |
| Toilet type | **Public toilet** | Public Toilet: 35.6% |
|  | Flushing toilet facility | Flushing Toilet: 32.8% |
|  | “Other” toilet facility | No facility: 5.4%  Pit Latrine: 8.8%  KVIP (ventilated): 14.3%  Bucket/pan: 2.5%  Other: 0.5% |
| Dwelling type | **Rooms in a compound house** | Rooms in Compound House: 58.7% |
|  | Separate house, semi-detached, or flat/apartment | Separate House: 15.4%  Semi-detached House: 8.4%  Flat/Apartment: 6.7% |
|  | “Other” dwelling type | Hut/Building: 0.9%  Tent: 0.2%  Improvised Home: 6.1%  Living Quarters Attached to Shop: 0.8%  Uncompleted Building: 2.6%  Other: 0.3% |
| Wall Materials | **Cement/Concrete walls** | Cement/Concrete: 83.6% |
|  | “Other” walls | Mud Brick/Earth: 1.8%  Wood: 10.7%  Metal Sheet: 1.2%  Stone: 0.3%  Burnt Bricks: 0.4%  Landcrete: 0.3%  Bamboo: 0.1%  Palm Leaves/Raffia: 0.1%  Other: 1.6% |
| Roof Materials | **“Other” roof type** | Mud/earth: 0.3%  Wood: 0.9%  Slate/asbestos: 43.1%  Cement or concrete: 3.9%  Roofing tiles: 1.5%  Bamboo: 0.2%  Thatch/raffia: 0.4%  Other: 0.7% |
|  | Metal sheet roof | Metal Sheet: 49.1% |
| Floor Materials | **Cement/Concrete floor materials** | Cement/Concrete: 80.0% |
|  | “Other” floor materials | Mud/Earth: 4.8%  Stone: 0.5%  Burnt Bricks: 0.2%  Wood: 3.9%  Vinyl Tiles: 2.7%  Ceramic/Marble/Granite: 4.0%  Terrazzo Flooring: 3.5%  Other: 0.4% |
| Cooking Fuel Type | **Solid fuel for cooking** | None, No Cooking: 7.0%  Wood: 2.1%  Charcoal: 45.1%  Crop Residue: 0.1%  Saw Dust: 0.3%  Animal Waste: 0.1%  Other: 0.3% |
|  | Clean fuel for cooking | Gas: 42.9%  Electricity: 1.0%  Kerosene: 1.2% |
| Rubbish Disposal | **Collected** | Collected: 50.7% |
|  | Public dump | Public Dump (Container): 26.3%  Public Dump (Open Space): 7.2% |
|  | “Other” rubbish disposal | Burned: 11.9%  Dumped Indiscriminately: 1.7%  Buried: 1.2%  Other: 0.9% |

**Table A2.** Variables used in regression models calculated directly at the EA-level in urban GAMA using remote sensing or census data.

| Variable | Calculation | Data source |
| --- | --- | --- |
| Overcrowding in home | Average household person to bedroom ratio (household size/# of bedrooms) in each EA (standardized) | Ghana 2010 Census |
| Population density | Number of individuals for each EA divided by area of that EA (in km^2^) (standardized) | Shapefile provided by the GSS |
| Elevation | Elevation above sea level in each EA minus the mean elevation of a 5km buffer around each EA (standardized) | DEM raster data provided by NASA |
| NDVI (vegetation abundance) | Average NDVI score in each EA (standardized) | Landsat-8 raster data from USGS EarthExplorer |

**Table A3.** Cross validation for model selection. Reported MSE of predicted values against fitted values for deprived area probabilities, with various samples removed for prediction.

| **Number of EAs removed for prediction (% of overall sample)** | **Model 1 (no random effects) MSE** | **Model 2 (independent random effects) MSE** |
| --- | --- | --- |
| 60 (2.5%) | 0.0013 | 0.066 |
| 90 (3.7%) | 0.0047 | 0.079 |
| 120 (5.0%) | 0.0029 | 0.080 |
| 150 (6.2%) | 0.0011 | 0.079 |
| 180 (7.4%) | 0.0010 | 0.065 |
| 210 (8.7%) | 0.0017 | 0.069 |
| 240 (9.9%) | 0.0002 | 0.073 |
